# Incidence and Clinical Outcomes of Hypothyroidism in Patients Undergoing Spinal Fusion

**DOI:** 10.7759/cureus.17099

**Published:** 2021-08-11

**Authors:** Evan Luther, Roberto J Perez-Roman, David J McCarthy, Joshua D Burks, Jean-Paul Bryant, Karthik Madhavan, Steven Vanni, Michael Y Wang

**Affiliations:** 1 Neurological Surgery, University of Miami Miller School of Medicine, Miami, USA; 2 Neurosurgery, University of Pittsburgh Medical Center, Pittsburgh, USA; 3 Department of Neurosurgery, Sanford Health, North Dakota, USA

**Keywords:** hypothyroidism, spine fusion, spondylosis, cardioprotection, mortality, acute myocardial infarction

## Abstract

Background

Hypothyroidism has been independently associated with the development of several comorbidities and is known to increase complication rates in non-spinal surgeries. However, there are limited data regarding the effects of hypothyroidism in major spine surgery. Therefore, we present the largest retrospective analysis evaluating outcomes in hypothyroid patients undergoing spinal fusion.

Methods

A retrospective review of the National Inpatient Sample (NIS) from 2004-2014 was performed. Patients with an International Classification of Diseases, 9th revision, Clinical Modification (ICD-9-CM) procedure code indicating spinal fusion (81.04-81.08, 81.34-81.38, 81.0x, 81.3x) were included. Patients with an ICD-9-CM diagnosis code indicating hypothyroidism (244.x) were compared to those without. Cervical and lumbar fusions were evaluated independently. Significant covariates in univariable logistic regression were utilized to construct multivariable models to analyze the effect of hypothyroidism on perioperative morbidity and mortality.

Results

A total of 4,149,125 patients were identified, of which 9.4% were hypothyroid. Although, hypothyroid patients had a higher risk of hematologic complications (lumbar - odds ratio [OR] 1.176, p < 0.0001; cervical - OR 1.162, p < 0.0001), they exhibited decreased in-hospital mortality (lumbar - OR .643, p < 0.0001; cervical - OR .606, p < 0.0001). Hypothyroid lumbar fusion patients also demonstrated decreased rates of perioperative myocardial infarction (MI) (OR .851, p < 0.0001). All these results were independent of patient gender.

Conclusions

Hypothyroid patients undergoing spinal fusion demonstrated lower rates of inpatient mortality and, in lumbar fusions, also had lower rates of acute MI when compared to their euthyroid counterparts. This suggests that hypothyroidism may offer protection against all-cause mortality and may be cardioprotective in the postoperative period for lumbar spinal fusions independent of patient gender.

## Introduction

The population of the United States has been increasing over the last several decades and, in turn, the prevalence of chronic illnesses has risen. Therefore, it is not surprising that the incidence of degenerative spine disorders in the United States (US) has also continued to grow. Although fusion techniques have improved markedly since their inception, the identification and prevention of comorbidities that predispose patients to perioperative complications remain limited. Yet, as our population continues to age and thus continues to develop and live with chronic illnesses, perioperative harm reduction becomes ever more imperative. Diabetes mellitus (DM), obesity, systemic malignancy, and cardiovascular disease are all conditions associated with an increased risk of perioperative adverse events in spine surgery [1]. However, hypothyroidism, which carries a prevalence of approximately 1%-4% in the US and has been independently associated with the development of hypertension, cardiovascular disease, osteoporosis, peripheral neuropathy, muscular weakness, and increased complication rates in non-spinal surgeries, has had only a limited number of studies suggesting that it confers increased perioperative risk in patients undergoing major spine surgery [2-10]. Therefore, the purpose of this study was to confirm the hypothesis that hypothyroidism negatively affects perioperative morbidity and mortality in this patient population.

## Materials and methods

Data source

Data were extracted from the National Inpatient Sample (NIS), the largest publicly available all-payer inpatient database in the US, between the years 2004 and 2014 [11]. The NIS is sponsored by the Agency for Healthcare Research and Quality as part of the Healthcare Cost and Utilization Project and is a 20% stratified sample of US hospital admissions for which weighted estimates characterize more than 95% of all inpatient US hospitalizations in any given year [11]. Furthermore, this database has previously been validated as a means to retrospectively review patients undergoing spinal surgery in the US.

Inclusion criteria

Patients who had an International Classification of Diseases, 9th revision, Clinical Modification (ICD-9-CM) procedure code indicating spinal fusion (81.04-81.08, 81.34-81.38, 81.0x, 81.3x) were included. Admissions with the following diagnoses were excluded: neoplasms (140.x-239), intraspinal abscess (324.1), pregnancy-related diagnoses (630-679.xx), inflammatory spondyloarthropathies (720.xx), osteomyelitis outside of the spine (730, 730.0, 730.01-730.07, 730.1, 730.11-730.17, 730.2, 730.21-730.27, 730.3x, 730.7x, 730.8, 730.81-730.87, 730.9, and 730.91-730.97), pathologic vertebral fractures (733.1, 733.10, 733.13, and 733.95), non-union or mal-union of vertebral fractures (733.8x, 733.9, 733.90-733.92, 733.95 and 733.99), vertebral fractures with or without spinal cord injury (805.xx and 806.xx), all vertebral dislocations (839 - 839.5x), and all vehicular accidents (E800.x - E849.x). This was done in an effort to only evaluate patients undergoing spinal fusion for spondylotic disease. Admissions with a ICD-9-CM diagnosis code indicating hyperthyroidism (242.xx) were also excluded to prevent confounding bias.

Covariates

Patient age, sex, race, year of admission, comorbidities, hospital location, hospital size, hospital teaching status, weekend admission status, elective admission status, diagnoses, procedures performed, and primary expected payer were extracted as independent variables. Comorbidity scoring was calculated using the Elixhauser et al. method [12]. Hypothyroidism, identified by the ICD-9-CM diagnosis code 244.x, was also extracted as an additional independent variable.

Outcome measures

Thirteen outcomes were evaluated: inpatient mortality; length of hospital stay; discharge disposition; neurologic complications, such as iatrogenic stroke and transient mental disorders such as delirium (997.0x and 293.xx); respiratory complications such as respiratory failure, pneumonia, and need for re-intubation (518.4-518.53, 518.81-518.84, 997.3x, 480-486, 96-96.05, and 96.7x); acute myocardial infarction (MI) (410.xx and 997.1); hematologic complications including postoperative anemia/thrombocytopenia, need for transfusion and subsequent transfusion reactions (285.1, 287.4x, 999.4-999.79, 999.8, 999.80, 999.83-999.89, and 99.0x); gastrointestinal (GI) complications, including acute gastritis, cholecystitis, and pancreatitis (535.0x, 570, 575.0, 577.0, 997.4, and 997.49); acute kidney failure (AKI) (584.x and 997.5); wound complications, such as postoperative hematoma, seroma, wound dehiscence, soft tissue infection, and osteomyelitis (998.1x, 998.3-998.32, 998.5x, 998.83, 999.3, 730.00, 730.08-730.09, 730.10, 730.18-730.19, 730.20, 730.28-730.29, 730.80, 730.88-730.89, 730.90, and 730.98-730.99); hardware complications, such as pseudoarthrosis (996.4x, 996.6, 996.60, 996.66-996.67, 996.69, 996.7, 996.70, and 996.78-996.79); and intraoperative durotomy (349.3x). Pulmonary embolism (PE) and deep venous thrombosis (DVT) (415.1x, 453.4x, and 997.2) were also evaluated as a separate outcome measure independent from other respiratory complications.

Statistical analysis

Data are presented as mean and standard deviation for continuous variables, and as frequency for categorical variables. Statistics of means were carried out using the unpaired Student's t-test, with and without equal variance (Levene’s test), and the Wilcoxon rank-sum tests when variables were not normally distributed. Statistics of frequencies were carried out with the chi-squared or Fischer exact tests as necessary. Patient, hospital, and Elixhauser comorbidity covariates were utilized in logistic regression analyses to assess associations between hypothyroidism and the previously mentioned outcome measures. Covariates predictive in univariable analysis (p<0.20) were entered into a multivariable model, which was refined with backward elimination. Statistical significance was achieved for p < 0.05 in multivariable regression analyses. Cervical and lumbar fusions were evaluated independently. Statistical analysis was performed with SAS 9.4 (SAS Institute, Cary, NC).

## Results

A total of 4,149,125 patients underwent spinal fusion in the US from 2004-2014 of which 389,970 (9.4%) also carried the diagnosis of hypothyroidism. Cervical and lumbar fusions were evaluated independently given the inherent differences in surgical approach and postoperative management.

Cervical

A total of 1,681,805 patients (40.5%) were identified as having undergone cervical spinal fusion during the time period in question. Their average age was 53.7 years (SD 27.0), and 51.7% of the patients were female. A total of 138,495 (8.2%) carried the diagnosis of hypothyroidism. The most common form of patient payment was private insurance (54.2%), followed by Medicaid (27.3%), and a significant majority of the patients were treated in the southern United States (43.4%, p<0.0001). The most common primary diagnoses were cervical disc displacement without myelopathy (31.8%), cervical spondylosis with myelopathy (16.0%), and cervical spondylosis without myelopathy (14.4 %). The average length of stay (LOS) was 2.4 days (SD 8.8).

When compared to their euthyroid counterpart, hypothyroid patients were significantly older (euthyroid 53.3 ± 26.9, hypothyroid 58.4 ± 26.2; p<0.0001), and females were found to have a significantly higher rate of hypothyroidism (12.6% of females vs. 3.7% of males; p<0.0001). LOS was significantly longer in hypothyroid patients (2.3 vs. 2.6 days; p<0.0001), and they also exhibited a lower rate of home discharge (81.7% vs. 87.4%) with associated higher rates of discharge to intermediate care facilities or need for home health (17.8% vs.12.03%, p<0.0001) (Table 1).

**Table 1:** Age, Gender, Length of Stay, Discharge Disposition, and Elixhauser Comorbidity Index for Hypothyroid Patients Undergoing Cervical and Lumbar Fusions *Elix = Elixhauser

	Cervical (n=1,681,805)				Lumbar (n=2,467,320)		
	Hypothyroid (n=138,495)	Euthyroid (n=1,543,310)	P-value		Hypothyroid (n=251,475)	Euthyroid (n=2,215,845)	P-value
Age, mean ± SD	58.4 ± 26.2	53.3 ± 26.9	< 0.0001		63.1 ± 27.5	54.1 ± 36.9	< 0.0001
Female	107,424 (78.4)	747,444 (49.3)	< 0.0001		203,478 (81.8)	1,170,334 (53.7)	< 0.0001
Discharge Disposition			< 0.0001				< 0.0001
Home	111,874 (81.7)	1,326,561 (87.4)			138,750 (55.8)	1,524,920 (69.9)	
Death	159 (0.12)	2315 (0.15)			234 (0.09)	2618 (0.12)	
Home Health	86733 (5.7)	11174 (8.2)			44,416 (17.9)	321,475 (14.7)	
Intermediate facility	95720 (6.3)	13194 (9.6)			63,527 (25.6)	320,034 (14.6)	
Elix. readmission index*	6.15 ± 19.0	3.99 ± 15.9	< 0.0001		7.35 ± 20.3	4.96 ± 17.3	< 0.0001

Lastly, hypothyroid patients, independent of gender, had a higher likelihood of hematologic (OR 1.162, 95% CI 1.114-1.212, p < 0.0001), hardware (OR 1.057, 95% CI 1.025-1.091, p < 0.0005), wound (OR 1.088, 95% CI 1.023-1.156, p < 0.0066), and neurologic complications (OR 1.125, 95% CI 1.039-1.216, p < 0.002). However, they also exhibited a large reduction in in-hospital mortality (OR .606, 95% CI .506-.72, p < 0.0001). There were no significant differences in rates of respiratory or GI complications, AKI, acute MI, PE/DVTs, or intraoperative durotomies (Table 2).

**Table 2:** Perioperative Mortality and Morbidity for Hypothyroid Patients Undergoing Cervical and Lumbar Fusion MI: myocardial infarction; GI: gastrointestinal AKI: acute kidney injury; PE: pulmonary embolism; DVT: deep venous thrombosis

	Cervical				Lumbar		
	Odds Ratio	95% CI	P-value		Odds Ratio	95% CI	P-value
Mortality	0.61	0.51-0.72	< 0.0001		0.643	0.55-0.75	< 0.0001
Neurologic complication	1.13	1.04-1.22	0.0033		0.919	0.88-0.96	< 0.0001
Respiratory complication	NS	NS	NS		NS	NS	NS
Acute MI	NS	NS	NS		0.851	0.81-0.89	< 0.0001
Hematologic complication	1.16	1.11-1.21	< 0.0001		1.176	1.16-1.19	< 0.0001
GI complication	NS	NS	NS		NS	NS	NS
AKI	NS	NS	NS		NS	NS	NS
DVT/PE	NS	NS	NS		NS	NS	NS
Wound complication	1.09	1.02-1.16	0.0066		NS	NS	NS
Hardware complication	1.06	1.03-1.09	0.0005		1.067	1.05-1.09	< 0.0001
Durotomy	NS	NS	NS		1.051	1.02-1.08	0.0002

Lumbar

A total of 2,467,320 patients (59.5%) were identified as having undergone lumbar spinal fusion during the time period in question. Their average age was 55 years (SD 36.5) and 56.6% of the patients were female. A total of 251,475 (10.19%) carried the diagnosis of hypothyroidism. The most common form of patient payment was private insurance (43.3%), followed by Medicaid (35.7%), and a significant majority of the patients were treated in the southern United States (40.8%; p<0.0001). The most common primary diagnoses were lumbosacral intervertebral disc degeneration (20.6%), displacement of a lumbar intervertebral disc without myelopathy (15.5%), and spinal stenosis with neurogenic claudication (14.9%). The average LOS was 2.4 days (SD 8.8).

Similar to the cervical cohort, the hypothyroid patients undergoing lumbar fusion were significantly older (euthyroid 54.1 ± 36.9, hypothyroid 63.1 ± 27.5; p<0.0001), and females had a significantly higher rate of hypothyroidism (14.8% of females vs. 4.3 % of males; p<0.0001). LOS was significantly longer in hypothyroid patients (4.0 vs 4.3 days; p<0.0001), and they also exhibited a lower rate of home discharge (55.8% vs. 69.9%) and higher rates of discharge to intermediate care facilities or need home health (43.4% vs. 29.4%; p<0.0001) (Table 1). These hypothyroid patients, again independent of gender, were also at a higher likelihood of hematologic (OR 1.176, 95% CI 1.160 to 1.192, p < 0.0001) and hardware complications (OR 1.067, 95% CI 1.048-1.087, p < 0.0001). They also exhibited decreased in-hospital mortality rates (OR .643, 95% CI .551 to .746, p < 0.0001), but unique to the hypothyroid lumbar fusion patients were their decreased rates of perioperative MI (OR .851, 95% CI .810 to .893, p < 0.0001) and neurologic complications (OR .919, 95% CI .884-.955, p < 0.0001). There were no significant differences in rates of respiratory, GI, or wound complications, or AKI and PE/DVTs. However, they did demonstrate increased rates of intraoperative durotomy (OR 1.051, 95% CI 1.023-1.079, p < 0.0002) compared to the euthyroid lumbar fusion patients (Table 2).

## Discussion

In recent years, endocrinopathies have become more recognized as modifiable risk factors associated with increased perioperative complication rates. For example, DM has been studied extensively and elevated hemoglobin A1c has been identified as an independent risk factor associated with increased hospital stays, elevated overall healthcare costs per individual, and higher rates of perioperative respiratory, cardiac, and genitourinary complications for various surgery types [3,13-14]. Hypothyroidism represents one of the most common endocrine disorders, with an estimated prevalence between 0.6 and 12 per 1000 in women and between 1.3 and 4.0 per 1000 in men [15]. Although several studies have linked hypothyroidism to an increase in perioperative complications for various surgical procedures, there remains limited data regarding the impact of hypothyroidism on major spine surgery [5-6,8-9].

In 2010, Walid et al. analyzed the effects of endocrine disorders on hospital LOS and found that hypothyroid patients who underwent lumbar decompression and fusion had a mean LOS 1 day longer than their euthyroid counterparts [14]. Our study reflects these results demonstrating an increased LOS of .3 days for hypothyroid patients in lumbar fusions. However, their study was limited in size with only 787 patients and thus found no change in LOS for cervical fusions. In contrast, our study also demonstrated an increased mean LOS of .3 days in the hypothyroid group who underwent a cervical fusion.

In 2015, Akins et al. first identified hypothyroidism as an independent risk factor (OR 1.29, 95% CI: 1.01-1.64, p < 0.041) for 30-day readmission after instrumented spine surgery [16]. However, a stratified analysis evaluating cervical and lumbar fusions separately was not performed thus introducing confounding bias to the study. Our study used the Elixhauser readmission index to determine the likelihood of 30-day readmission for patients with hypothyroidism and found results that agreed with those of Akins; in both cervical and lumbar fusions, hypothyroid patients had higher indices suggesting they had a higher likelihood of 30-day readmission (Table 1).

In 2018, Vakharia et al. published the first study evaluating complications directly associated with hypothyroidism in the first 90 days post-single level anterior cervical discectomy and fusion (ACDF). They found that hypothyroid patients had higher rates of non-healing wounds (OR 2.27, 95% CI: 1.11-4.62, p < 0.0232), PE (OR 2.16, 95% CI: 1.51-3.09, p < 0.001), MI (OR 2.10, 95% CI: 1.43-3.08, p < 0.001), and 90-day readmission (OR 1.45, 95% CI: 1.41-1.49, p < 0.001) [17]. Although our study also showed increased rates of wound complications in cervical fusions, we elected to include all types of cervical fusions, both posterior and anterior, in an effort to eliminate the selection bias that limited their analysis. Moreover, they utilized a database that is generated from a single-payer, and only 107,066 patients were identified between 2005 and 2014. Furthermore, posterior cervical fusions and multilevel ACDFs were excluded. These limitations generated a significant amount of selection bias because patients from a single insurer may not accurately represent the US population with hypothyroidism and the surgical approach chosen for any given procedure tends to be surgeon- and patient-dependent and therefore does not directly imply presenting pathology type or surgical indication [17]. Lastly, they chose to perform a matched case-control analysis, which can be technically complicated and is wrought with the possibility of improper statistics [18]. Both of these studies also did not report mortality rates whatsoever. Therefore, to our knowledge, this is the first comprehensive analysis of the effects of hypothyroidism on perioperative morbidity and mortality in patients undergoing cervical or lumbar spinal fusion.

In our series, the prevalence of hypothyroidism was 9.4%, more than double the reported prevalence in the US [4,7]. This may be explained by the fact that thyroid hormone (TH) is a known mediator of bone metabolism and normal serum levels are essential for proper skeletal growth and preservation of bone mass, a lack of which, may ultimately lead to degenerative changes making hypothyroid patients more prone to requiring corrective spine surgery [19-20]. In this study, hypothyroidism was also associated with higher rates of postoperative anemia or thrombocytopenia, need for transfusion or associated transfusion reactions, and pseudoarthrosis for both cervical and lumbar fusions. In lumbar fusions, there was also an increased likelihood of intraoperative durotomy and in cervical fusions, there was an increased likelihood of wound complications such as hematoma, seroma, infection, and dehiscence as well as an increased rate of iatrogenic neurologic injury. These results appear to be consistent with prior reports that hypothyroidism increases perioperative complication rates. However, and most interestingly, hypothyroid patients in both cervical and lumbar fusions exhibited decreased in-hospital mortality, and in lumbar fusions, they also had decreased rates of perioperative MI and iatrogenic neurologic injury. Dhital et al. reported similar results using the NIS to evaluate the effects of hypothyroidism on patients with coronary heart disease (CHD). They found that although patients with hypothyroidism were at higher risk of having CHD (OR 1.11, 95% CI: 1.09-1.12, p < 0.001), they were, in fact, at lower risk of developing acute MI (OR .71, 95% CI: .70-.72, p < 0.001), and in those with acute MI, hypothyroidism was found to decrease in-hospital mortality (OR .86, 95% CI: .83-.88, p < 0.001) [11]. These results suggest that a more robust understanding of the pathophysiologic changes seen in hypothyroid patients is necessary to better elucidate those attributes responsible for cardioprotection and decreased mortality in this population.

It is well-known that important cardiovascular and metabolic changes occur secondary to lower levels of serum TH; decreased response to alpha-1 stimulation increases systemic vascular resistance, decreased nitric oxide availability reduces vasodilation resulting in endothelial dysfunction, and altered lipid metabolism creates increased levels of circulating low-density lipoproteins all of which can lead to higher rates of CHD and DM [8,11,21-25]. Therefore, the question then remains how any of these mechanisms could lead to a reduction in mortality and acute MI. There is conflicting data on whether hypothyroidism induces a hyper- or hypocoagulable state, but good evidence exists to suggest that hypothyroidism does, in fact, accelerate atherosclerosis [11,26-28]. This chronic increase in atherosclerotic plaque development ultimately leads to increases in coronary vessel stenosis, and it is this long-term exposure to sublethal ischemia that is believed to provoke the development of a robust collateral circulation that ultimately protects the heart from future acute MIs [11,29]. However, the association between hypothyroidism and decreased mortality rates is more poorly understood.

Although our study draws strength from its utilization of a large nationwide database that records patient information from many insurers and is thus intended to accurately represent the annual US inpatient population, it remains retrospective in nature and the NIS provides no information regarding post-discharge course or readmissions [11]. Furthermore, with any sample size as large as the one in this study, statistically significant differences may be identified but remain difficult to interpret clinically especially if their odds ratio remains close to 1. Also, any analyses performed on NIS data rely on accurate ICD-9-CM coding, which is inherently error-prone [17,30]. Lastly, the NIS provides no information on duration or severity of diagnosis, current medical therapies, patient compliance with said therapies, and thyroid hormone levels. As a result, only correlations between disease states can be identified without true proof of causality [11].

## Conclusions

Hypothyroidism has been identified as a modifiable risk factor leading to increased perioperative complications in various surgical procedures, yet there remains conflicting evidence regarding its effects on spinal fusions. This study adds to the growing body of literature suggesting that although hypothyroidism may predispose patients to certain complications, it may also provide cardioprotection and decreased mortality rates in certain populations. This study highlights the need for prospective trials to evaluate the mechanistic effects of TH levels on various tissue types in surgical patients. Until this is clarified, few recommendations can be made to improve perioperative clinical outcomes.
